# Subcortical Contribution to the Role of the Basal Ganglia in Action Selection

**DOI:** 10.2174/1570159X2209240229143211

**Published:** 2024-02-29

**Authors:** Veronique Coizet, Frederic Ambroggi

**Affiliations:** 1 Univ. Grenoble Alpes, Inserm, U1216, CHU Grenoble Alpes, Grenoble Institut Neurosciences, 38000 Grenoble, France;; 2 Aix-Marseille Université, CNRS, Laboratoire de Neurosciences Cognitives, UMR 7291, Marseille, France;; 3 Institut de Neurosciences de la Timone: Aix-Marseille Univ, CNRS, INT, Marseille, France

   The basal ganglia are one of the fundamental processing units of the vertebrate brain. As such, they have evolved multiple connections with most regions of the cerebral cortex, limbic system, thalamus, and numerous structures in the hindbrain. Despite the suggestions that the basal ganglia are involved in a large range of functions, it is now widely accepted to evidence an underlying role of this network in basic selection processes and goal-directed behavior [1-3]. The anatomy and function of the basal ganglia have been extensively studied in relation to the cortex. Connections between the cerebral cortex and basal ganglia can be viewed as a series of parallel projecting loops or channels [4, 5]. Much experimental evidence now supports the concept that cortico-basal ganglia-thalamocortical channels have an important anatomical and functional significance [4-6]. However, prior to the evolutionary expansion of the cerebral cortex, it was probably the coevolution of the basal ganglia with subcortical structures that first established a basic circuitry onto which the cortex was later grafted [7]. Therefore, the concept of potentially segregated parallel projecting loops through the basal ganglia has been extended to their connections with sensorimotor and motivational structures in the brainstem, including the superior colliculus, periaqueductal gray, pedunculopontine and parabrachial nuclei [8], and the thalamus. This subcortical network has long been underestimated and understudied, while recent innovative studies highlight their contribution to the role of the basal ganglia in action selection. This special issue presents selected reviews and research articles to illustrate this contribution.

   Frost-Nylen and colleagues first give a brief overview of the basic design of the basal ganglia, which remained very similar since the lamprey diverged from the evolutionary line leading to mammals some 500 million years ago. The number of each type of neuron has increased manifold in parallel to the richness of the increasing behavioral repertoire. This review describes the microcircuitry within each nucleus of the basal ganglia and the control the basal ganglia can exert on the activity of the downstream midbrain and brainstem.

   However, the interactions between the basal ganglia and the midbrain/brainstem are also reciprocal. Ascending brainstem/midbrain projections significantly influence information processed by the basal ganglia. Vautrelle and colleagues, as well as Zhan and Reynolds, demonstrate how the cortical input to striatal medium spiny neurons and cholinergic interneurons are modulated by sensory inputs from the brainstem. Bradfield and colleagues propose that the interactions between acetylcholine and dopamine in the striatum represent the neuronal level by which situation-appropriate action selection takes place *via* mesencephalic, thalamic and cortical inputs. Sicre and colleagues show that the activity of parafascicular thalamic neurons is compatible with that found in striatal cholinergic interneurons and suggest this circuit could repress the engagement in action according to the motivational level of the animal. Finally, the mesencephalic dopaminergic input to the caudal globus pallidus external part *via* D2 receptors, is suggested by Espallergues and colleagues to optimize the selection, initiation and execution of actions.

   If the cortex and the thalamus have been classically considered as the main source of information toward the basal ganglia, recent work highlights a more dynamic role of the brainstem inputs to this network. For example, Melleu and Canteras, as well as Coizet and colleagues, review how the connections between the superior colliculus, the parabrachial nucleus and the periaqueductal gray nucleus toward the basal ganglia are suggested to take part of action selection processes in approach and defense behavior. Zhang and colleagues also demonstrate that the control of dopamine neurons by the pedunculopontine GABA neurons participates in the modulation of goal-directed behavior in behavioral tasks with positive and negative valence. The mesencephalic locomotor subregions are proposed by Margentern and Esposita to represent independent entities providing multiple locomotor subcircuits with the basal ganglia to participate in the execution and tuning of behavior. Finally, Esposito and colleagues present an overview of the recent literature highlighting the role of the mesencephalic-basal ganglia closed loops in modulating implicit selective attention.

   Altogether, these articles emphasize a more complex functional and anatomical link to the basal ganglia, involving both cortical and subcortical structures, which should incentivize additional research on the diversity of these interrelations.
